# Strengthening Healthcare Through Research: The Academic Evolution of Indus Hospital & Health Network

**DOI:** 10.12669/pjms.42.(ICON26).15976

**Published:** 2026-04

**Authors:** Shaukat Ali Jawaid

For the past several years, *Pakistan Journal of Medical Sciences* has been collaborating with various medical institutions, both nationally and internationally, to promote a research culture by providing them with an opportunity to publish special supplements showcasing the scholarly work undertaken by their faculty. In the past, we have collaborated with institutions such as Isfahan University of Medical Sciences in Iran, Wuhan Institute of Technology in China, Indus Hospital & Health Network in Pakistan, and Punjab Institute of Neurosciences at Lahore. Our most recent collaboration was with the Department of Surgery at Aga Khan University Hospital at Karachi.

This is the third supplement we have published for Indus Hospital & Health Network, scheduled for release in April 2026 on the eve of their 8th Biennial Conference (ICON 26). Previously, supplements were published in 2022 with the theme *“Beyond the Call of Duty”* and in 2024 with the theme *“Game-Changing Solutions in Healthcare.”*

The current supplement includes nineteen original articles, six case reports, two letters, and two editorials. All manuscripts underwent initial screening, editorial triage, comprehensive internal and external peer review, as well as plagiarism checks before acceptance for publication. To ensure quality and maintain standards, numerous manuscripts required multiple rounds of revision.

In 2021, we conducted workshops on scientific writing and publication ethics for the faculty and support staff of Indus Hospital involved in this project. Subsequently, detailed sessions were held from time to time with those responsible for coordinating the supplement to brief them on journal requirements. It was clearly emphasized that all studies must receive approval from the Ethics Committee and Institutional Review Board, that ICMJE authorship guidelines must be strictly followed, and that gift authorship—an unethical practice amounting to intellectual corruption—must be avoided at all costs.

We strictly adhere to publication ethics and follow the guidelines of the Committee on Publication Ethics (COPE UK), of which the journal is a member. This collaboration also provides authors and researchers an opportunity to learn and update themselves on the latest developments in medical journalism.

However, challenges were encountered. Members of the coordinating committee at Indus Hospital are frequently replaced, and some appeared not to take responsibilities seriously. As a result, many submissions had to be returned for revision and some were rejected. Common deficiencies included references not cited in proper order, incomplete bibliographic details, and citation of articles published in predatory journals—indicating a lack of awareness about paper mills and predatory publishing practices. Authors were instructed to remove or replace such references before further processing.

Two manuscripts were rejected due to excessive plagiarism, with similarity scores exceeding 30 percent. Another issue noted was reluctance on the part of some authors to submit manuscripts through the journal’s online submission system. If authors do not submit their manuscripts themselves, they will not learn to use the Open Journal System effectively. While some studies included in the supplement represent high-quality research, others required substantial revision before being considered suitable. In one case, the study objectives were not clearly stated, yet the manuscript was forwarded in that incomplete form—reflecting a non-serious attitude that is incompatible with scientific writing.

Despite these challenges, we are pleased that the final supplement contains several valuable studies that will likely increase citations from Pakistan and enhance the country’s contribution to global medical literature. It will also further strengthen the academic image and credibility of Indus Hospital & Health Network.

Some of the important studies included in this supplement are summarized below:

Syed Ghazanfar Saleem and colleagues, in their study on multivessel disease and total coronary occlusion, concluded that a significant subset of NSTEMI patients in Pakistan present with underlying occlusive myocardial infarction (OMI). Simple clinical and ECG features may help identify such high-risk cases in resource-limited settings, enabling timely intervention and improved outcomes.^3^

Muhammad Hammad Ajmal et al., in their study on the frequency of deep vein thrombosis in patients undergoing total knee replacement using a pneumatic tourniquet, reported that despite routine thromboprophylaxis and tourniquet use, the incidence of DVT among the fifty-three patients studied did not increase—likely due to careful patient selection and early postoperative rehabilitation.^4^

In a time-motion analysis of patient flow in a family medicine department, Humayoon Khalid Saeed and colleagues identified significant delays in diagnostic and pharmacy processes. The authors suggested system-level reforms, particularly increasing diagnostic capacity, to optimize workflow.^5^

Sahrish Haji et al., exploring timelines and consistency in critical hematology results reporting, concluded that rapid and reliable communication of critical values is essential for timely clinical intervention and improved patient outcomes. They have recommended staff training, automated alert systems, and continuous performance monitoring to enhance accuracy and efficiency.^6^

Nourina and colleagues selected the important topic of health literacy and its associated factors. Their findings indicate that among Pakistani youth, higher health literacy is strongly influenced by educational attainment, parental literacy—particularly maternal literacy—and active online health information seeking. They recommend educational and digital health interventions to improve long-term health outcomes.^7^

Poonam Devi et al., in their study on pre-donation deferral patterns in blood donor recruitment, highlighted challenges including a high proportion of first-time donors and gender disparities. They suggest targeted measures to address risk factors and improve donor retention, thereby strengthening blood transfusion services in low- and middle-income countries.^8^

Munawar Sarwar Ali and colleagues have discussed the scope, training, and systemic challenges of Family Medicine in South Asia. They have concluded that despite some progress, the specialty remains constrained by limited capacity building and weak career recognition. Strengthening academic departments, postgraduate programs, and career pathways is essential to advance primary healthcare in the region.^9^

Another noteworthy study examined traditional practices and beliefs regarding placental disposal in Pakistan. The authors noted that these practices have not been adequately incorporated into safe clinical protocols, creating gaps in environmental safety. They advocate for culturally sensitive, environmentally sustainable, and climate-resilient approaches within healthcare systems.^10^

Sana Ehsan Ullah and colleagues conducted a randomized controlled comparison of Sublay and Onlay mesh techniques in ventral abdominal wall hernia repair. They reported that Sublay mesh repair significantly reduces wound infection compared to Onlay repair, with comparable seroma and recurrence rates, supporting its preference in resource-limited settings like Pakistan.^11^

Overall, this supplement represents an excellent academic effort by the faculty of Indus Hospital & Health Network to showcase their research contributions. We at *Pakistan Journal of Medical Sciences* are pleased to have supported this unique healthcare institution in Pakistan, which provides free healthcare services. Prof. Abdul Bari Khan and his colleagues deserve commendation for their dedication and leadership. We wish them continued progress and success in their mission.
